# Increasing reliability of axially compressed cylinders through stiffness tailoring and optimization

**DOI:** 10.1098/rsta.2022.0034

**Published:** 2023-04-03

**Authors:** R. L. Lincoln, P. M. Weaver, A. Pirrera, R. M. J. Groh

**Affiliations:** Bristol Composites Institute, University of Bristol, BS8 1TR Bristol, UK

**Keywords:** buckling, stiffness tailoring

## Abstract

The capabilities of the rapid tow shearing (RTS) process are explored to reduce the well-known imperfection sensitivity of axially compressed cylindrical shells. RTS deposits curvilinear carbon fibre tapes with a fibre-angle-thickness coupling that enables the *in situ* manufacturing of embedded rings and stringers. By blending the material’s elastic modulus and wall thickness smoothly across the cylindrical surface, the load paths can be redistributed favourably with a minimal-design approach that contains part count and weight while ameliorating imperfection sensitivity. A genetic algorithm that incorporates realistic manufacturing imperfections and axial stiffness penalty is used to maximize the 99.9% reliability load of straight fibre (SF) and RTS cylinders. The axial stiffness penalty ensures that reliability does not come at the expense of stiffness. The first-order second-moment method is used to calculate statistical moments that enable an estimate of the 99.9% reliability load. Due to the fibre-angle-thickness coupling of RTS, buckling data are normalized by mass and thickness. Compared to a quasi-isotropic laminate, which corresponds to the optimal eight-layer design for a perfect cylinder, the optimized SF and RTS laminates have a 6% and 8% greater 99.9% normalized reliability load. By relaxing the axial stiffness penalty, the performance benefit can be increased such that SF and RTS cylinders exceed the 99.9% normalized reliability load of an eight-layer quasi-isotropic laminate by 23% and 37%, respectively. Both improvements (with and without penalty functions) stem largely from a reduction in the variance of the buckling-load distribution, thereby demonstrating the potential of fibre-steered cylinders in reducing the imperfection sensitivity of cylindrical shells.

This article is part of the theme issue ‘Probing and dynamics of shock sensitive shells’.

## 1. Introduction

Thin-walled cylinders are highly efficient monocoque shells used within the aerospace, civil and energy sectors, among others. However, the designers of axially compressed thin-walled shells must contend with the disparity between theory and experiment as collapse due to the loss of stability often occurs at levels well below the predicted buckling load. Von Kármán & Tsien [1] demonstrated that the post-buckling of a cylinder is dominated by an unstable equilibrium path stemming from a subcritical bifurcation and, therefore, the cylinder is sensitive to imperfections in the pre-buckling regime. The sensitivity to imperfections was quantified by Koiter [2] in his PhD thesis, whereby small geometric imperfections (of the order of a wall thickness) were shown to reduce the buckling load dramatically.

The buckling phenomenon can be understood qualitatively from an energy perspective. When a cylinder is compressed, the strain energy of the system increases through the addition of membrane energy. The system is initially stable in this unbuckled state representing a global energy minimum. On the pre-buckling path, an isotropic axially compressed cylinder has numerous energetically favourable pathways to buckling [3]. That is, in the equilibrium manifold that describes the totality of the energy paths the cylinder could take, there are many competing paths that favourably exchange membrane energy for bending energy (buckling). However, as the strain energy of the system increases, the energy landscape evolves and a new global minimum, corresponding to a buckled shape, is more energetically favourable. Interestingly, even when the cylinder is still nominally in the pre-buckling state, there exists another disconnected (in axial load vs. axial compression space) equilibrium state, with just a small energy barrier separating the two regimes. A transition over this so-called mountain-pass point [4] represents the lowest energy pathway that exchanges membrane energy for bending energy and induces buckling. Physically, the mountain pass point materializes as a single dimple on the cylinder surface. Indeed, there are multiple mountain pass points in the energy landscape, one between the pre-buckling state and each of the many possible post-buckling states. However, the mountain pass point represented by the single dimple is the smallest of such mountain pass points once it exists (for a certain applied end-shortening). Due to the rotational invariance of an isotropic cylinder, the initiation of a single dimple can occur at any circumferential location and is strongly dependent on the precise nature of existing initial imperfections. This phenomenon is described as ‘spatial chaos’ [3,5–9] due to its analogy to temporal chaos whereby initial conditions drastically affect loading trajectories through time.

To alleviate some of the aforementioned sensitivity to initial imperfections of axially compressed isotropic shell, stiffening elements (stringers and rings) are often used. Stiffened, a.k.a. semi-monocoque, shells reduce imperfection sensitivity by breaking the cylinder surface into effective curved panels [10]. This can be explained as follows. Firstly, the reinforcement adds stiffness, which increases the energy required for initiation of lateral deflection and for the loss of stability. Secondly, in addition to retarding the onset of a buckle, stiffeners cause a transition of the critical buckling mode to intra-panel buckling, i.e. the panel traps the lateral buckling mode. The latter mode is not only geometrically confined but, more importantly, inherently super-critical and, hence, less sensitive to imperfections. The use of stringers and rings in cylindrical shells is standard practice in thin-walled structural design as, for given load carrying capability, semi-monocoque shells are lighter than their unstiffened counterparts. However, the integration of stiffening elements is resource intensive. Typically, two stages are required to combine the outer skin and the stiffeners, increasing time-to-manufacture and cost, and increasing locations of possible failure (e.g. stiffener debonding or cracks in weld lands). Despite these drawbacks, the uncertainty of imperfection-sensitive monocoque cylinders leads designers to choose stiffened shells. However, recent advances in modern composite manufacturing techniques offer the opportunity to design inherently imperfection-insensitive cylinders. These advanced manufacturing techniques create variable angle tow (VAT) composite parts, where fibre paths are curvilinear, in contrast to straight in their straight fibre (SF) equivalent. By arranging fibre paths in a curved way, the stiffness field, and therefore load path, of an axially compressed cylinder is not uniform across the surface. This non-uniformity breaks the symmetry of the problem very much like stiffeners do, with similarly positive consequences.

**Figure 1.**
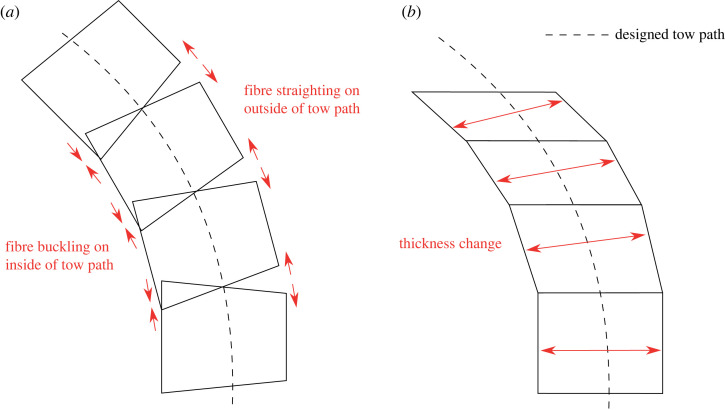
Variable angle tow shearing techniques: (*a*) automated fibre placement (AFP) and (*b*) rapid tow shearing (RTS). AFP bends the tow along the designed tow path, potentially causing fibre buckling and straightening on the inside and outside of the tow, respectively. RTS shears the tow with respect to the designed tow path, increasing the local thickness and decreasing local width. RTS can tessellate adjacent tows whereas AFP cannot. (Online version in colour.)

Of all possible VAT technologies, the present work focuses on using rapid tow shearing (RTS)—a derivative of continuous tow shearing with greater deposition rate—developed by Kim *et al.* [11] to overcome the manufacturing defects commonly encountered in automated fibre placement (AFP). AFP, the most common VAT manufacturing technique, developed in the 1980s [12], places curvilinear fibre paths through in-plane bending of a fibre tow as shown in figure 1*a*. As the distance of each fibre in the tow to the central reference path is different, fibre buckling and straightening can occur on the concave and convex side of the tow, respectively [13], as shown in figure 1*a*. The mismatch in curvature also eliminates the ability to tessellate adjacent tows, leading to gaps and overlaps that are known to play a significant part in the failure of AFP components [14]. In contrast to AFP, RTS (shown in figure 1*b*) shears tows in the plane of placement, creating a uniform radius of curvature across the tape and enabling perfect tessellation of adjacent tows.

In addition to the defect-free manufacturing ability of RTS, it has an interesting geometric feature: fibre-angle-thickness coupling. As a tow is sheared, the local thickness increases perpendicular to the fibre path according to a secant relationship such that the local thickness of the kth RTS lamina in a stack is given by

1.1 $$t^{k}=\frac{t_{0}^{k}}{\cos(\gamma^{k})},$$

where $t^{k}$ is the local sheared lamina’s thickness, $t_{0}^{k}$ is the nominal thickness of the unsheared lamina and $\gamma^{k}$ is the local lamina shearing angle. The limit to the shearing angle is set by defect-free manufacturing constraints to be $70^{\circ }$ [15]. The thickness build-up adds another dimensionality to the design of fibre-reinforced cylinders; embedded stiffeners can now be created by shearing the tow periodically, opening up the possibility to embed hoops, stringers, orthogrids and isogrids within the structure during manufacturing.

Previous numerical [16,17] work has shown that RTS-designed cylinders exhibit reduced sensitivity to geometric imperfections compared to SF cylinders. While the EI contribution of the embedded stiffeners is not large enough to create panel-breaking elements, the EI contribution does provide enough local stiffness to tailor the structural response of the cylinder. To corroborate these results, an RTS and a quasi-isotropic (QI) cylinder were manufactured and tested in axial compression [18]. In the experiment, the RTS cylinder had a 10% greater buckling load than the QI cylinder [18]. Furthermore, finite-element (FE) simulations indicate that, if manufacturing imperfections had been equal across both cylinders, the RTS cylinder would have a 10% greater mass-specific buckling load than the QI cylinder. The present work builds on this previous research by optimizing towards an imperfection-insensitive cylinder through reliability-based optimization and stiffness tailoring.

Optimization studies of fibre angles for variable-stiffness plates have demonstrated significant weight-savings due to enhanced mechanical properties in pre- and post-buckling [19–21]; reducing stress concentrations around cutouts [22]; and improving buckling of sandwich panels [23]. Variable-stiffness cylinders have been optimized for bending [24,25], buckling [26,27] and fundamental vibration frequency [28] using linear analyses. However, limited work has investigated the optimization of fibre angle of variable-stiffness cylinders to maximize the nonlinear buckling load in the presence of geometric imperfections. As imperfections are known to reduce the load-carrying capacity of cylindrical shells, an optimization that accounts for imperfections while maximizing buckling load could lead to a more imperfection-insensitive design.

For example, Lindgaard *et al.* [29] optimized the fibre angles of an SF cylinder for nonlinear buckling while considering a superposition of eigenmodes as imperfections. The researchers considered this imperfection signature to be the ‘worst’ as it is ‘an imperfection shape which yields the lowest limit load’ [29]. However, the ‘worst’ imperfection is known to be the single dimple [4] as it is the lowest energy pathway between pre-buckling and post-buckling regimes. However, both single dimple and eigenmode-affine imperfections are not necessarily realistic imperfections in composite cylinder manufacture, as composite cylinders are typically dominated by low-wavenumber periodic modes [30,31].

As imperfections in thin-walled cylinders are stochastic, Bolotin [32] suggested the use of probabilistic analyses to capture the variability of buckling response. A Fourier series representation of measured geometric imperfections is often used as these have been shown to capture the features of realistic imperfection signatures [33]. However, to accurately model a realistic imperfection requires a significant number of Fourier coefficients which can be untenable for probabilistic methods, such as a Monte Carlo analysis. Elishakoff [34] suggested a semi-analytical framework to overcome the computational cost of Monte Carlo analyses referred to as a first-order second-moment (FOSM) method. By using a first-order approximation of the Taylor series expansion of the reliability function to estimate FOSM statistics (mean and variance, respectively), the reliability function can be approximated [35–37]. In this way, the computational cost of reliability-based, probabilistic analyses is reduced and can be implemented in an optimization.

To the authors’ knowledge, limited work has been conducted on the optimization of SF or tow-sheared composite layups to maximize a conservative buckling estimate of imperfect-geometry cylinders with realistic imperfections, i.e. a buckling load in the left tail of the predicted buckling load distribution. The present manuscript addresses this gap within the literature by optimizing the fibre angles for maximal 99.9% reliability load of SF and tow-sheared cylinders with realistic geometric imperfections derived from a dataset of measured composite cylinders [35]. Furthermore, a penalty function is applied to ensure that the axial stiffness of the cylinder is comparable to a defined baseline cylinder design.

The remainder of paper is structured as follows. Section 2 details the nomenclature and theory of the RTS manufacturing technique. Section 3 describes the FOSM methodology, including estimations of mean and variance, and description of imperfection signatures. Section 4 covers the formulation of the optimizations for the SF and RTS cylinders. Section 5 details the results of the optimizations for the SF and RTS cylinders. Conclusions and areas of future work are summarized in §6.

## 2. Rapid tow sheared cylinder nomenclature

To define an RTS lamina, we adapt Gürdal & Olmedo’s [38] well-known nomenclature, such that a fibre path is defined by

**Figure 2.**
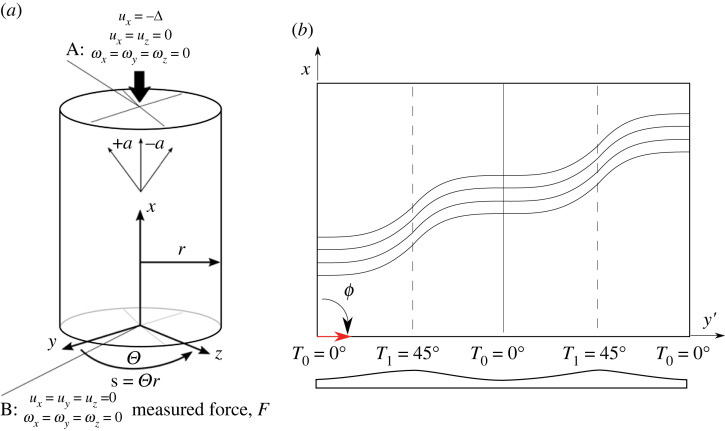
(*a*) Coordinate system to define cylinder geometry and boundary conditions used for the FE analyses. Fibre angles are measured counter-clockwise from the global x-axis. Point A corresponds to a multi-point constraint between a reference point at the centroid of the top end of the cylinder and all nodes at the top edge of the cylinder. Five degrees of freedom are constrained at A and only the axial direction, $u_{x}$, is free to enforce a displacement, −Δ. Point B is a multi-point constraint between a bottom central reference point and all bottom-edge nodes. All 6 d.f. are constrained at B. The reaction force of the cylinder is measured at B. (*b*) A single tow-sheared lamina with the nomenclature as defined in equation (2.1). (Online version in colour.)

2.1 $$\phi {\left\langle T_{0}|T_{1}\right\rangle }^{n},$$

where ϕ defines the direction of the tow-placement head (the steering direction), $T_{0}$ defines the initial shearing angle offset from ϕ, $T_{1}$ defines the shearing angle in the middle of a shearing period, and n is the shearing periodicity across the length or circumference of the cylinder.A $T_{0}\to T_{1}\to T_{0}$ cycle is one period and it is assumed that the variation in fibre angle between $T_{0}$ and $T_{1}$ is linear. The direction of the tow-placement head, ϕ, is nominally measured clockwise from the global *x*-axis (here taken to be the axis of the cylinder), $T_{0}$ and $T_{1}$ are nominally measured counter-clockwise from the ϕ axis. For SF laminates, fibre angles are measured counter-clockwise from the global *x*-axis. The coordinate system used herein is shown in figure 2*a*.

An application of the nomenclature is shown in figure 2*b* for a $90{\left\langle 0|45\right\rangle }^{2}$ lamina. The cross-section shows that the shearing process creates embedded stiffeners perpendicular to the ϕ direction as a result of the RTS shearing-thickness coupling. In this instance, two embedded stringers are created due the periodicity n=2. By combining $\phi =0^{\circ }$ and $\phi =90^{\circ }$ plies in a laminate, orthogrid-like structures can be manufactured. For the interested reader, further information on the RTS manufacturing process can be found in [11,15,39,40].

## 3. First-order second-moment methodology

In the present manuscript, the first-order second-moment (FOSM) method is not described in detail and interested readers are referred to [34,35,37]. However, the motivation and applicability of the FOSM analysis is discussed in §3a and the modelling steps involved are listed in §3b.

### (a) Motivation and applicability

It is helpful to appreciate the motivation and applicability behind using the FOSM method: a desire to calculate a specific reliability point by estimating statistical features (mean and variance) of an unknown distribution of buckling loads with minimal analyses. To achieve this, the FOSM analysis includes two simplifications: the distribution of buckling loads calculated is assumed to be normal and the set of imperfections that are superimposed on the cylinder geometry follows a predefined and non-changing statistical signature (constant covariance matrix). The assumption that the buckling load distribution is normal is, strictly speaking, not accurate as the distribution of limit loads generally follows an extreme value distribution [8,41], but the assumption of normality is often made in the literature. Indeed, the distribution of buckling loads may change throughout an optimization but these considerations are outside of the scope of the present work. It is also worth noting that throughout the optimization process, cylinder designs with low variance are automatically chosen as these designs satisfy the objective function of increased reliability. In this scenario of low variance, extreme value distributions of buckling loads can be approximated by a normal distribution. As a result, in preliminary work we have determined that Monte Carlo simulations of optimized high reliability designs are generally well approximated by a normal distribution. The second simplification, the use of a static set of imperfections, negates the fact that different layups have different imperfection signatures due to the curing and layup processes. However, without manufacturing simulations of each layup, it is impossible to account for the characteristic imperfection signatures that are present in each cylinder. Furthermore, a comparison between the Fourier coefficients of the mean imperfection used within the present FOSM analysis [35] and previously manufactured RTS and SF cylinders [18] indicates that the Fourier coefficients used are representative of real, manufactured structures.

The final feature of the FOSM analysis that enables the efficient calculation of statistical features is the Mahalanobis transformation. The Mahalanobis transformation can be understood in light of the probabilistic analysis: it is desirable to minimize the number of variables and to ensure the random variables are independent of one another. The random variables for the present analysis are the 462 Fourier coefficients that describe the imperfection field of the measured set of composite cylinders [35]. It is these random variables that are used within the FOSM analysis and are changed into uncorrelated values through the Mahalanobis transformation.

### (b) Description of the first-order second-moment method

**Figure 3.**
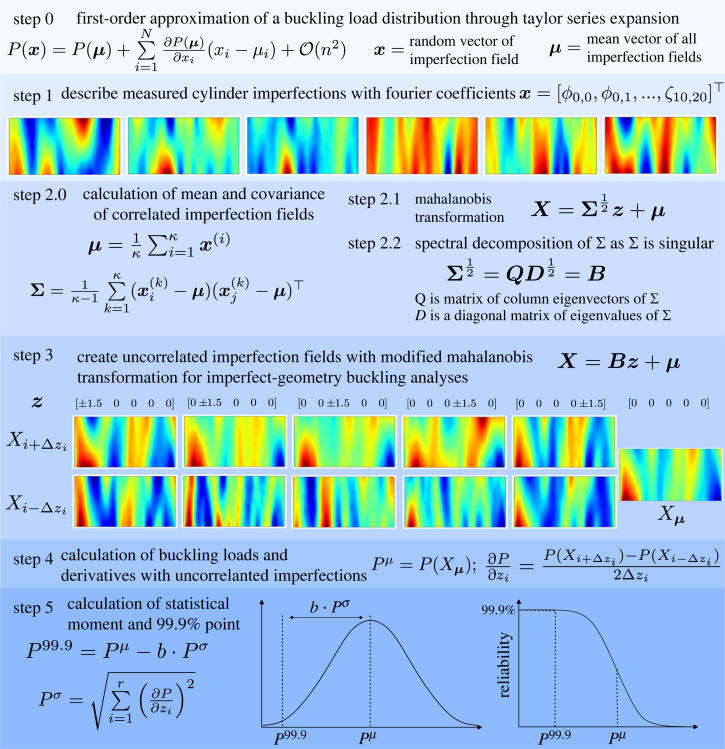
Schematic of the FOSM methodology and implementation of the Mahalanobis transformation. (Online version in colour.)

In light of the motivation and applicability of the FOSM method, the following description outlines the steps taken to calculate a high-reliability point across a distribution of buckling loads of thin-walled cylinders as shown in figure 3.

Step 0 The probability distribution of the buckling loads, assumed to be normal (as discussed in §3a), is approximated through a first-order approximation of a Taylor series expansion. Higher-order terms are neglected. The first-order derivatives are calculated through Steps 1–4.
Step 1 Six cylinder imperfection profiles (shown under Step 1 of figure 3) from real, measured cylinders are taken [35]. The measured imperfection signatures are described in terms of 11 axial and 21 circumferential Fourier coefficients (ϕ and ζ). The 462 by 462 variance-covariance matrix of these correlated variables is populated.
Step 2.0 The first step of the Mahalanobis transformation is to calculate the mean vector and variance-covariance matrix of the imperfection.
Step 2.1 The Mahalanobis transformation is used to remove correlation between the Fourier coefficients.
Step 2.2 To calculate the root of the variance-covariance matrix, a spectral decomposition is used as the variance-covariance matrix is singular.
Step 3 The modified Mahalanobis transformation is now used to create a set of uncorrelated imperfection fields derived from the principal components of the variance-covariance matrix and the mean imperfection signature.
Step 4 Each imperfection field calculated in Step 3 is applied to the cylinder design, and the imperfect-geometry buckling load is calculated with the FE solver (§4a) for each of the 11 imperfections. Then, a central-difference numerical derivative based on the imperfect-geometry buckling loads is calculated to find the first derivative of the buckling load distribution with respect to the uncorrelated variables. The mean buckling load is also calculated as the buckling load of the cylinder with the mean imperfection signature.
Step 5 The numerical derivatives are used to calculate the standard deviation of the buckling load distribution. A reliability point set to capture 99.9% of the cumulative normal distribution is used to calculate a high-reliability buckling load.

## 4. Optimization formulation

Primarily, the objective of the optimization is to converge on cylinder layups that maximize the imperfect-geometry buckling load. However, as the RTS process has an angle-to-thickness coupling, as discussed in §2, an RTS laminate has a thickness equal to or greater than the unsheared, SF equivalent. Thus, the mass of an RTS cylinder with shearing is greater than an SF laminate. To remove the influence of mass on the buckling load, the mass-specific buckling load can be calculated as $\tilde{P}=P/m$, where P is the buckling load and m is the mass of the cylinder. Nominally, the linear buckling load of an isotropic cylinder [42] given by

4.1 $$P=\frac{2E\pi t^{2}}{\sqrt{3(1-\nu^{2})}},$$

shows there is quadratic relationship between buckling load and thickness—that is $P\propto t^{2}$. As m∝t, there is still a first-order relationship between mass-specific buckling load and thickness: $\tilde{P}\propto t$. Therefore, the mass-specific buckling load is normalized by thickness so that

4.2 $$\hat{P}=\frac{\tilde{P}}{\bar{t}},$$

where $\hat{P}$ is the thickness-normalized buckling load and $\bar{t}$ is the average thickness of the laminate. In this way, the RTS laminates and SF laminates can be compared directly as the influence of thickness has been removed. Within the present manuscript the use of the terminology ‘buckling load’ refers to the limit load of the structure: the point at which the structure loses stability. Both the theoretical linear critical load (shown in equation (4.1)) and the limit loads of the imperfect structures discussed in §§4 and 5 are referred to herein as the perfect-geometry buckling load and imperfect-geometry buckling load, respectively.

The layup of the SF cylinder to be optimized is a double angle-ply laminate. Two angles are used in the eight-layer laminate as it enables a comparison to the RTS laminate, a double ply-pair eight-layered laminate. The RTS laminate is a double ply-pair laminate to compare to a previously manufactured RTS cylinder [18]. The SF layup is

4.3 $${\left[\pm \alpha_{1},\pm \alpha_{2}\right]}_{\text{s}}\text{, }$$

such that the formulation of the SF optimization is

4.4 $$\begin{array}{rl} \max_{x}\; & {\hat{P}}^{99.9}(x)\cdot d(x)\\ \text{variables}\; & x=[\alpha_{1},\alpha_{2}]\\ \text{s.t.}\; & 0\leq \alpha_{i}\leq 90\;(i=1,2)\\ \; & d(x)=\min{\left(1,\frac{E_{\text{SF}}}{E_{\text{QI}}}\right)}^{3}, \end{array}$$

where the exponent 3 in the axial stiffness penalty, d(x), is chosen from a trial-and-error analysis. The purpose of penalty function is to remove low axial stiffness designs from the gene pool but not have a strict threshold for axial stiffness. A strict threshold would have limited the possibility of a high imperfect-geometry buckling load with, say, 95% of the axial stiffness of a QI laminate. $E_{\text{SF}}$ and $E_{\text{QI}}$ are the axial stiffness of the SF and QI cylinder, respectively, calculated as

4.5 $$E=\frac{PL}{2\pi r\bar{t}u}\text{, }$$

where P is the imperfect-geometry buckling load, L is the length of the cylinder, r its radius, $\bar{t}$ its average wall thickness and u the applied displacement at the onset of buckling. The layup of the RTS cylinder to be optimized is

4.6 $${\left[\phi_{1}\pm {\left\langle T_{0_{1}}|T_{1_{1}}\right\rangle }^{n_{1}},\phi_{2}\pm {\left\langle T_{0_{2}}|T_{1_{2}}\right\rangle }^{n_{2}}\right]}_{\text{s}}\text{, }$$

where $\phi ,T_{0},T_{1}$ and n have their previous meanings as described in §2. The optimization is formulated for the RTS cylinder as follows:

4.7 $$\begin{array}{rl} \max_{x}\; & {\hat{P}}^{99.9}(x)\cdot d(x)\\ \text{variables}\; & x=[\phi_{1},T_{0_{1}},T_{1_{1}},n_{1},\phi_{2},T_{0_{2}},T_{1_{2}},n_{2}]\\ \text{s.t.}\; & \phi_{i}=\{0,90\}\;(i=1,2)\\ & 0\leq T_{j_{i}}\leq 70\;(j=0,1,i=1,2)\\ & \text{when }\phi_{i}=0,\;n_{i}=0,1,2,\ldots ,10\;(i=1,2)\\ & \text{when }\phi_{i}=90,\;n_{i}=0,1,2,\ldots ,18\;(i=1,2)\\ & d(x)=\min{\left(1,\frac{E_{\text{RTS}}}{E_{\text{QI}}}\right)}^{3}, \end{array}$$

where $E_{\text{RTS}}$ is the axial stiffness for the RTS cylinder and all other variables have their previous meanings. The fibre angles for the SF and RTS cylinder are always integer values to reflect the accuracy of the RTS method [15].

A genetic algorithm (GA) is used to optimize both SF and RTS cylinders. The optimization of fibre angles of composite laminates is known to be a non-convex design problem: results are sensitive to starting points and convergence is not guaranteed [43]. By defining the composite structure with lamination parameters instead of fibre angles the design space can be transformed into a convex design problem under certain conditions [44–47]. A second optimization step is then required to translate the lamination parameters into a manufacturable layup of fibre angles. For only two unique layers in the laminate stacking sequence (balanced and symmetric layup), the lamination parameter approach is not expected to be faster than an optimization based on fibre angles directly. Hence, this work conducts the optimization in fibre angle design space. For verification of the GA, the SF optimized result is compared against an exhaustive search. In addition, the GA was verified with benchmark functions. Both SF and RTS optimizations have a population of 30 and run for 30 generations. For each generation, the number of elite children is 2, the crossover and mutant fractions were 70% and 30% of the remaining population, respectively, reflecting similar values to literature [48]. Python [49] scripting is used to pre-process input files, such as defining element-by-element fibre angles, post-process results, and execute the GA.

### (a) Geometrically nonlinear imperfect-geometry buckling analyses

The values of $\hat{P}$ and E are calculated using the commercial FE solver Abaqus [50]. S4R elements with enhanced hourglassing control are used with the mesh size informed by a convergence study. For a discussion on the discretization of fibre angles and thicknesses for RTS structures, the reader is directed to [51]. The optimization is carried out on SF- and RTS-designed cylinders with geometry and material properties as listed in table 1. For all analyses, wagon-wheel type boundary conditions are used, shown in figure 2*a*. The wagon-wheel boundary conditions are implemented with a multi-point constraint between a central control node and the circumferential nodes on the top or bottom of the cylinder. The top reference node is constrained in 5 d.f., with the axial direction (*x*-axis) left free for application of the axial displacement, $u_{x}=-\Delta$. The bottom reference node is constrained in all six degrees of freedom and the reaction force, F, from the applied displacement, is measured at the reference point. Wagon-wheel type boundary conditions are a standard approach of modelling cylinder buckling in an FE setting [53].

**Table 1.** Geometry of cylinder and material properties of carbon fibre pre-preg IM7/8552 [52]. Lamina level material properties are given with a nominal fibre volume fraction of 60%. The nominal thickness $t_{0}$ refers to all eight plies at the unsheared thickness.

r	L	$t_{0}$	$E_{11}$	$E_{22}$	$\nu_{12}$	$G_{12}$	$G_{13}$	$G_{23}$	ρ
(mm)	(mm)	(mm)	(MPa)	(MPa)	—	(MPa)	(MPa)	(MPa)	(${g\;\mathrm{mm}}^{-3}$)
300	1040	1.048	138 171	9722	0.356	4900	4900	3352	$1.57\times 10^{-3}$

The imperfect-geometry buckling load of the cylinders is calculated using a geometrically nonlinear Static, General analysis in Abaqus. For each analysis, the imperfection signatures are implemented in the FE environment through the construction of an orphan mesh based on the number of circumferential and axial nodes. The step size of the incremental-iterative Newton solver is monitored until the solver fails to converge, indicating a limit point instability. The numerical convergence of an imperfect-geometry cylinder is driven primarily by the minimum step size allowed within the Newton solver: the smaller the allowable step size the closer the solver approaches the limit point instability. However, the smaller the allowable step size, the longer the analysis takes. We therefore seek a trade-off between computational accuracy and time. A step-size study is carried out on 50 random RTS laminates to ensure that the selected step size is sufficient to calculate the imperfect-geometry buckling load accurately while balancing computational time.

**Figure 4.**
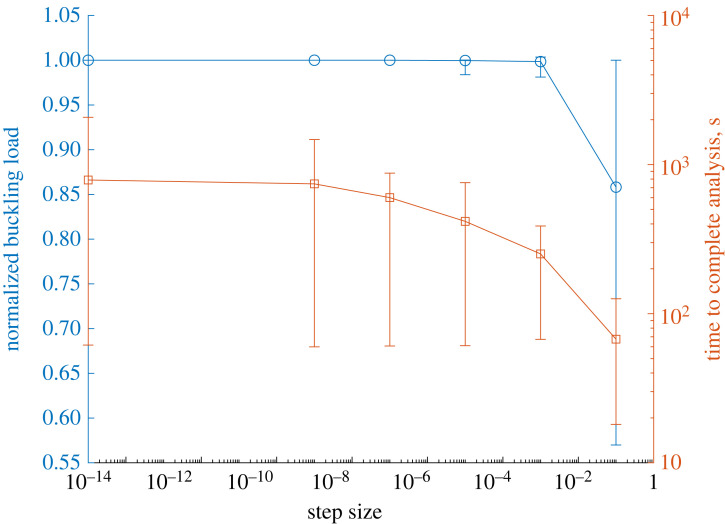
Step-size study for 50 random RTS laminates by comparing normalized buckling load and computational time. Normalization of the buckling load is done with respect to the $1\times 10^{-15}$ step size, taken to be most accurate. Data points for load and analysis time represent the arithmetic mean of 50 laminates. Error bars indicate maximum and minimum data points. (Online version in colour.)

The results of the step-size study are presented in figure 4, where the normalization of the imperfect-geometry buckling load is done with respect to the ‘most accurate’ results as calculated by the $1\times 10^{-15}$ step-size analysis. From figure 4, it is clear that the step size $1\times 10^{-3}$ represents a reasonable trade-off between computational time and accuracy. Thus, this value is used in all further geometrically nonlinear analyses. Quantitatively, the analyses with a step size of $1\times 10^{-3}$ have a normalized mean imperfect-geometry buckling load of 0.999 with a standard deviation of 0.003.^1^

## 5. Optimization results

The optimization was first run on the SF laminate. The ${\hat{P}}^{99.9}$ values for ply angle combinations are compared to a QI laminate, with a percentage change calculated with respect to the thickness-normalized 99.9% imperfect-geometry buckling load, $\Delta {\hat{P}}_{\text{QI}}^{99.9}$, defined as

5.1 $$\Delta {\hat{P}}_{\text{QI}}^{99.9}=100\cdot \frac{{\hat{P}}_{\text{lam}}^{99.9}-{\hat{P}}_{\text{QI}}^{99.9}}{{\hat{P}}_{\text{QI}}^{99.9}}\text{, }$$

where the subscripts lam and QI refer to the laminate considered and QI laminate, respectively.

The QI laminate is chosen as the reference for comparison because it has been shown to be the best SF laminate for perfect-geometry cylinder buckling [54]. The eight-layer QI, however, is not the overall optimal QI layup due to the deleterious influence of anisotropy [55], as when compared to a homogeneous and specially orthotropic 48-layered QI laminate (i.e. no anisotropy) with identical overall thickness, the eight-layer laminate has 89% of the imperfect-geometry buckling load of the 48-layer one.^2^ (The thickness of each ply in the 48-layer QI laminate was scaled to equal the wall thickness of the eight-layer QI.) Nevertheless, given the manufacturing implications of laminating a 48-layer cylinder, the eight-layer QI is taken to be the optimal SF laminate for perfect cylinder buckling herein. As both an eight-layer QI and eight-layer RTS cylinder have been manufactured and tested [18], we limit the number of plies to be eight to enable comparison between the laminates in this study.

### (a) Straight fibre cylinder results

The solution converged upon by the solver is described in table 2 as the laminate ${\mathrm{SF}}_{1}$ and has a ${\left[\pm 21,\pm 66\right]}_{\text{s}}$ layup. ${\mathrm{SF}}_{\text{1}}$ has a 5% greater ${\hat{P}}^{99.9}$ but a 7% lower ${\hat{P}}^{\mu }$ when compared to the QI laminate, indicating that the greater ${\hat{P}}^{99.9}$ is due to a reduced standard deviation, ${\hat{P}}^{\sigma }$. This is indeed the case, with ${\mathrm{SF}}_{\text{1}}$ having a 54% lower standard deviation than the QI laminate. It appears that in this optimization, the solver favoured layups with lower standard deviation rather than greater ${\hat{P}}^{\mu }$. The preference for decreased variance is driven by the high-reliability requirement of 99.9% and the b=3.0902 factor used in calculating ${\hat{P}}^{99.9}$. For example, a decrease in 1 kN/kg.mm in ${\hat{P}}^{\sigma }$ increases ${\hat{P}}^{99.9}$ by 3.1 kN/kg.mm. To explore the design landscape further, and verify the optimization result, it is informative to perform an exhaustive search of the laminate design space ${\left[\pm \alpha_{1},\pm \alpha_{2}\right]}_{\text{s}}$. We explore the SF design space with an exhaustive search as it is not possible with the RTS laminates due to the high-dimensionality of the RTS layup.

**Table 2.** Data of SF and RTS optimizations. Converged result for RTS and SF optimizations are ${\mathrm{RTS}}_{\text{1}}$ and ${\mathrm{SF}}_{\text{1}}$, respectively. ${\mathrm{SF}}_{\text{2}}$ is the maximum ${\hat{P}}^{99.9}$ from the exhaustive search for the SF laminate. ${\mathrm{SF}}_{\text{3}}$ is the maximum fitness value from the exhaustive search for the SF laminate. ${\mathrm{RTS}}_{\text{2}}$ is the maximum ${\hat{P}}^{99.9}$ from the RTS optimization. ${\mathrm{RTS}}_{\text{man}}$ is the manufactured cylinder from [18]. Fit is the fitness value of the layup as calculated from $\mathrm{Fit}={\hat{P}}^{99.9}\cdot d(x)$, where d(x) is calculated from the axial stiffness penalty of equations (4.4) and (4.7). Thickness-normalized results for the 99.9% reliability limit, mean imperfection signature and standard deviation are described by the superscripts 99.9, μ and σ, respectively. Axial stiffness, E, is calculated from equation (4.5) and percentage change, $\Delta {\hat{P}}_{\text{QI}}^{99.9}$, is calculated from equation (5.1).

		fit	${\hat{P}}^{99.9}$	${\hat{P}}^{\mu }$	${\hat{P}}^{\sigma }$	E	$\Delta {\hat{P}}_{\text{QI}}^{99.9}$
ID	layup		(kN/kg.mm)			(GPa)	(%)
QI	${\left[\pm 45,0,90\right]}_{\text{s}}$	37.7	37.7	47.4	3.13	52.7	—
${\mathrm{SF}}_{1}$	${\left[\pm 21,\pm 66\right]}_{\text{s}}$	39.6	39.6	44.1	1.45	54.8	5.04
${\mathrm{SF}}_{\text{2}}^{a}$	${\left[\pm 35,90_{2}\right]}_{\text{s}}$	14.7	46.8	50.3	1.16	35.9	23.0
${\mathrm{SF}}_{\text{3}}$	${\left[\pm 22,\pm 66\right]}_{\text{s}}$	40.1	40.1	44.8	1.50	53.2	6.37
${\mathrm{RTS}}_{\text{1}}$	${\left[90\pm {\left\langle 69|67\right\rangle }^{15},0\pm {\left\langle 11|35\right\rangle }^{10}\right]}_{\text{s}}$	40.6	40.6	43.1	0.82	53.4	7.69
${\mathrm{RTS}}_{\text{2}}^{a}$	${\left[90\pm {\left\langle 58|67\right\rangle }^{9},0\pm {\left\langle 64|64\right\rangle }^{0}\right]}_{\text{s}}$	27.0	47.7	51.8	1.33	43.6	37.4
${\mathrm{RTS}}_{\text{man}}$	${\left[0\pm {\left\langle 20|25\right\rangle }^{2},90\pm {\left\langle 35|25\right\rangle }^{9}\right]}_{\text{s}}$	36.3	40.3	46.5	2.01	50.9	6.36

${\;}^{a}$No axial stiffness penalty.

#### (i) Symmetric straight fibre cylinder exhaustive search

**Figure 5.**
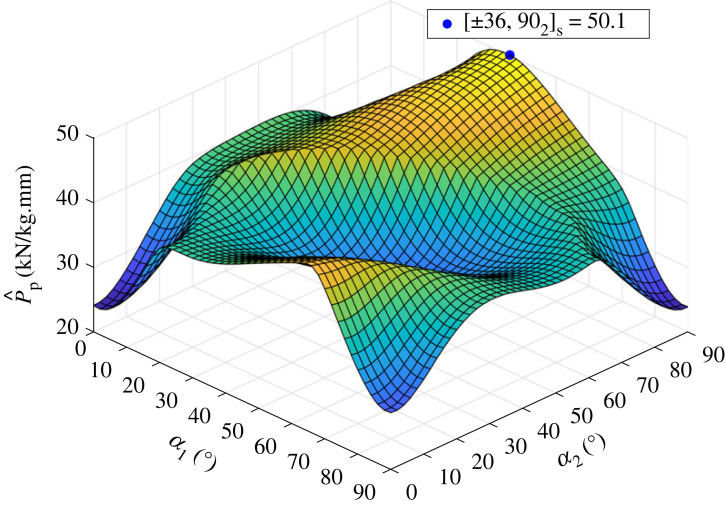
Landscape of the perfect-geometry, thickness-normalized imperfect-geometry buckling loads ${\hat{P}}_{\text{p}}\;\mathrm{for}\;{\left[\pm \alpha_{1},\pm \alpha_{2}\right]}_{s}$, where $\alpha_{1},\alpha_{2}\in [0,2,4,\ldots ,90]$. Blue dot is the global maximum ${\hat{P}}_{p}=50.1\;\mathrm{kN}/\mathrm{kg}.\mathrm{mm}$ for the layup ${\left[\pm 36,90_{2}\right]}_{s}$. (Online version in colour.)

An exhaustive search of the design landscape for the SF layup ${\left[\pm \alpha_{1},\pm \alpha_{2}\right]}_{\text{s}}$ for $\alpha_{1},\alpha_{2}\in [0,2,4,\ldots ,90]$ is conducted. First, the thickness-normalized perfect-geometry buckling load, ${\hat{P}}_{\text{p}}$, of the design landscape is investigated, as shown in figure 5. The maximal ${\hat{P}}_{\text{p}}$ is 50.1 kN/kg.mm in the laminate ${\left[\pm 36,\pm 90\right]}_{\text{s}}$, which is 14.5% lower when compared to the eight-layer QI with a ${\hat{P}}_{\text{p}}=58.6\;\mathrm{kN}/\mathrm{kg}.\mathrm{mm}$. The large valley in the centre of the response surface is attributed to a change in buckling mode shape between a doubly periodic and axisymmetric mode. The response surface has similarities to the unsymmetric laminate $\left[\pm \beta_{1},\pm \beta_{2}\right]$ investigated by Hühne [56] and Kriegesmann [57] which is discussed more fully in §5a(ii).

**Figure 6.**
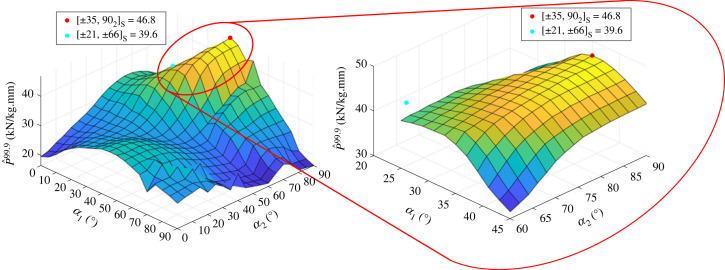
Coarse and fine mesh design landscape of the 99.9% reliability thickness-normalized buckling loads, ${\hat{P}}^{99.9}$, for ${\left[\pm \alpha_{1},\pm \alpha_{2}\right]}_{\text{s}}$ where $\alpha_{1},\alpha_{2}\in [0,5,\ldots ,90]$. Red dot is the global maximum ${\hat{P}}^{99.9}=46.8\;\mathrm{kN}/\mathrm{kg}.\mathrm{mm}$ for the layup ${\left[\pm 35,90_{2}\right]}_{\text{s}}$. Cyan dot is optimizer solution ${\mathrm{SF}}_{\text{1}}$ with ${\hat{P}}^{99.9}=39.6\;\mathrm{kN}/\mathrm{kg}.\mathrm{mm}$ for the layup ${\left[\pm 21,\pm 66\right]}_{\text{s}}$. Cyan and red dots refer to axial stiffness penalty and no axial stiffness penalty solutions, respectively. (Online version in colour.)

To investigate the imperfect-geometry response of the present structure, a search was conducted with respect to ${\hat{P}}^{99.9}$ for the ${\left[\pm \alpha_{1},\pm \alpha_{2}\right]}_{\text{s}}$ with $\alpha_{1},\alpha_{2}\in [0,5,\ldots ,90]$. The design landscape is shown in figure 6 with the global maximum, ${\mathrm{SF}}_{\text{2}}$, shown by the red dot and ${\mathrm{SF}}_{\text{1}}$ shown by the blue dot. The landscape is coarser than figure 5 as the number of FE analyses to calculate a central difference ${\hat{P}}^{99.9}$ for $2^{\circ }$ increment across ${\left[\pm \alpha_{1},\pm \alpha_{2}\right]}_{\text{s}}$ is 23276 compared to 3971 for a $5^{\circ }$ increment. An initial coarse-mesh analysis sweep of the landscape enabled a finer mesh analysis around the global optimum. The global maximum ${\hat{P}}^{99.9}$ is a ${\left[\pm 35,90_{2}\right]}_{\text{s}}$ laminate with a ${\hat{P}}^{99.9}=46.8\;\mathrm{kN}/\mathrm{kg}.\mathrm{mm}$, 23% greater than the QI laminate and 18.2% greater than ${\mathrm{SF}}_{\text{1}}$. The optimizer did not find laminate ${\mathrm{SF}}_{\text{2}}$ as it does not meet the axial stiffness penalty threshold and is therefore penalized during the optimization. As shown in table 2, the fitness value of ${\mathrm{SF}}_{\text{2}}$ is 14.7 kN/kg.mm due to the lower axial stiffness of the design and the heavy penalization due the penalty function d(x).

**Figure 7.**
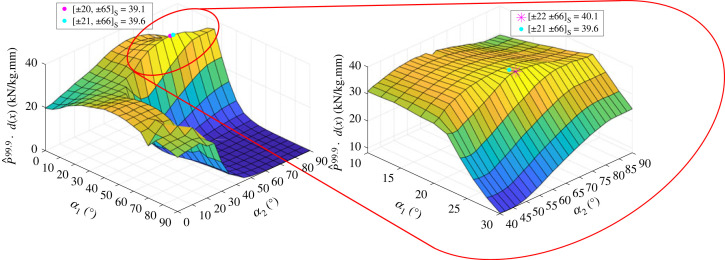
Coarse and fine mesh design landscape of the fitness (Fit) values $\mathrm{Fit}={\hat{P}}^{99.9}\cdot d(x)$ for ${\left[\pm \alpha_{1},\pm \alpha_{2}\right]}_{\text{s}}$, where $\alpha_{1},\alpha_{2}\in [0,5,\ldots ,90]$ and d(x) is the axial stiffness penalty described in equations (4.4) and (4.7). Magenta dot and star represent the global maximum Fit for the coarse and fine mesh, respectively. Maximum coarse mesh Fit =39.1 kN/kg.mm with layup ${\left[\pm 20,\pm 65\right]}_{s}$. Maximum fine mesh Fit =40.1 kN/kg.mm with layup ${\left[\pm 22,\pm 66\right]}_{s}$. Cyan dot is optimizer solution ${\mathrm{SF}}_{\text{1}}$ with Fit=39.6 kN/kg.mm for the layup ${\left[\pm 21,\pm 66\right]}_{s}$. (Online version in colour.)

The fitness response surface is depicted in figure 7 for both a coarse initial mesh and finer mesh in the region of interest. The large valley shown for $\alpha_{1},\alpha_{2}\geq 35^{\circ }$ is due to the axial stiffness penalty reducing the fitness of the design. The loss in axial stiffness occurs as the fibres are orientated circumferentially rather than axially. In the analysis of the fitness function, two optima were found based on the coarseness of the fibre angle distribution for the ${\left[\pm \alpha_{1},\pm \alpha_{2}\right]}_{\text{s}}$ layup. The first optimum found in the coarse mesh, a ${\left[\pm 20,\pm 65\right]}_{\text{s}}$ layup, has a fitness value of 39.1 kN/kg.mm. The second optimum found in the finer mesh, ${\mathrm{SF}}_{\text{3}}$, a ${\left[\pm 22,\pm 66\right]}_{\text{s}}$ layup, has a fitness value of 40.1 kN/kg.mm. For layups that have an axial stiffness equal to or greater than the QI, the fitness values are equal to the ${\hat{P}}^{99.9}$ of that laminate. The ${\hat{P}}^{99.9}$ of ${\mathrm{SF}}_{\text{3}}$ is 1.3% greater than the ${\hat{P}}^{99.9}$ of ${\mathrm{SF}}_{\text{1}}$, the design found by the optimizer, indicating that the optimization algorithm converged close to the optimal design. While the convergence of the SF optimization does not guarantee convergence for the RTS optimization, it indicates that the optimizer successfully converges *towards* high-reliability, thickness-normalized buckling loads.

#### (ii) Unsymmetric straight fibre cylinder exhaustive search

**Figure 8.**
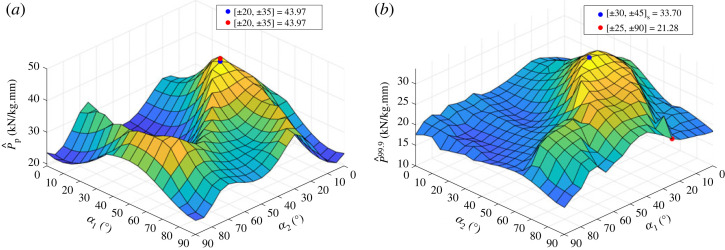
Response surface of unsymmetric laminate $\left[\pm \alpha_{1},\pm \alpha_{2}\right]$ for both (*a*) perfect-geometry linear buckling load and (*b*) imperfect-geometry 99.9% buckling load. All values of buckling load have been normalized by thickness and mass. Red dots indicate global optimums within the landscape explored, and red dots indicate the global optimums of Hühne [56] of the same laminate configuration. (Online version in colour.)

To compare to previous work on the perfect- and imperfect-geometry buckling load analysis of unsymmetric laminates [56,57], two exhaustive searches of the thickness-normalized buckling load are performed on the unsymmetric laminate $\left[\pm \alpha_{1},\pm \alpha_{2}\right]$. The first analysis is a perfect-geometry linear buckling analysis. The second is a FOSM analysis of the 99.9% buckling load of the imperfect-geometry laminate. The results of these two analyses are shown in figure 8*a*,*b* for the perfect- and imperfect-geometry cylinders, respectively. The optimal values (i.e. greatest values) have been indicated by the blue dots. The incrementation of each α was kept to $5^{\circ }$ to compare to Hühne’s results [56]. For the material system and geometry of cylinder investigated by Hühne, he found the optimal layup for: perfect-geometry buckling load to be [±20,±35], and for imperfect-geometry buckling load to be $\left[\pm 25,90_{2}\right]$. Both of Hühne’s optimal results are indicated by the red dots.

Figure 8*a* shows that for the geometry and material system under consideration, the global optimum of the perfect-geometry buckling load is the same as Hühne’s result, a [±20,±35] laminate. Given that geometric differences between the cylinders studied (the present cylinder is R=300 mm, t=1.048 mm and L=1040 mm, with Z≈3000, whereas Hühne’s cylinder is R=250 mm, t=0.5 mm and L=510 mm, and Z≈2000) is appreciable,^3^ it is interesting that the optimal layup for the perfect buckling load is the same. It has been shown that the optimal layup for cylinders of ‘intermediate size’ is independent of geometry [58] and this explains why the optimal unsymmetric layup for perfect buckling load is the same between Hühne’s results and the present analysis.

Figure 8*b* shows that the global optimum of the 99.9% buckling load of the unsymmetric laminate in the current material system and geometry, a [±30,±45] laminate, is not the same as Hühne’s result, a $\left[\pm 25,90_{2}\right]$ laminate. It is important to note that Hühne’s result plotted in figure 8*b* is for the maximum ‘$N_{15}$’: the greatest buckling load of the cylinders investigated with a perturbation load of 15 N applied radially at the midpoint of the cylinder exterior surface. As such, the red and blue dots shown in 8*b* have been derived by different methodologies. In the present work, the imperfect buckling load of a cylinder is derived from the 99.9% reliability load with a set of geometric imperfections, whereas in Hühne’s work, the imperfect buckling load is derived from a nominally perfect cylinder with a 15 N force perturbation force. Hence, the differences between the optimal layups derived are due to differences in methodology and also the aforementioned differences in geometry and material properties. In conclusion, both methodologies are valid and indicate that the means of deriving imperfect buckling loads must be explicit and map back to the design problem. For the present work, aiming to incorporate RTS-designed cylinders into rocket launch structures, the reliability of a design against realistic imperfections and high axial stiffness is key.

### (b) Rapid tow sheared cylinder results

The solution converged upon by the RTS solver, ${\mathrm{RTS}}_{\text{1}}$, is a ${\left[90\pm {\left\langle 69|67\right\rangle }^{15},0\pm {\left\langle 11|35\right\rangle }^{10}\right]}_{\text{s}}$ laminate with a ${\tilde{P}}^{99.9}=40.6\;\mathrm{kN}/\mathrm{kg}.\mathrm{mm}$, 7.69% greater than the eight-layer QI laminate and 1.2% greater than ${\mathrm{SF}}_{\text{3}}$. The optimizer has again converged on a laminate that minimizes variance with ${\hat{P}}^{\sigma }=0.82\;\mathrm{kN}/\mathrm{kg}.\mathrm{mm}$ for ${\mathrm{RTS}}_{\text{1}}$ being 73% lower than the eight-layer QI laminate and 43% lower than ${\mathrm{SF}}_{\text{1}}$. The orthogrid-type structure produced by ${\mathrm{RTS}}_{\text{1}}$ is similar to other RTS laminates that have high buckling loads [16] and the manufactured and tested RTS laminate, ${\mathrm{RTS}}_{\text{man}}$ [18]. The inner plies have shallow shearing angles, create embedded hoops and have an average thickness of 8% greater than the nominal ply thickness. It is interesting to note the high shearing angles of the first ply-pair of ${\mathrm{RTS}}_{\text{1}}$—the average thickness of the outer plies is 164% greater than the nominal thickness. However, despite the increase in average thickness, the thickness-normalized reliability buckling load is still greater than the QI laminate and SF laminates within the axially constrained optimization. As the difference between $T_{0}$ and $T_{1}$ is small, it appears that the optimizer has resulted in a uniformly thick outer ply-pair to increase its contribution to the second moment of area.

It is worth noting that some laminates within the optimization have greater ${\hat{P}}^{99.9}$ than the optimized results, but are penalized due to the axial stiffness penalty. The RTS cylinder with the greatest ${\hat{P}}^{99.9}$, ${\mathrm{RTS}}_{\text{2}}$ with layup ${\left[90\pm {\left\langle 58|67\right\rangle }^{9},0\pm {\left\langle 64|64\right\rangle }^{0}\right]}_{\text{s}}$, has ${\hat{P}}^{99.9}=47.7\;\mathrm{kN}/\mathrm{kg}.\mathrm{mm}$, which is 37.4% greater than the eight-layer QI cylinder. The ${\left\langle 64|64\right\rangle }^{0}$ lamina is a sheared, SF layer that has a thickness 128% greater than the nominal thickness. ${\mathrm{RTS}}_{\text{2}}$ has a lower fitness value than ${\mathrm{RTS}}_{\text{1}}$, as the axial stiffness is 17% lower than the eight-layer QI cylinder. However, between the SF and RTS cylinders without axial stiffness penalty, i.e. ${\mathrm{SF}}_{\text{2}}$ and ${\mathrm{RTS}}_{\text{2}}$, the RTS design achieves a better compromise between increased 99.9% reliability load and axial stiffness. While ${\mathrm{RTS}}_{\text{2}}$ improves on the QI 99.9% reliability load by 37.4% it only has a 17.3% reduction in axial stiffness, whereas ${\mathrm{SF}}_{\text{2}}$ loses 31.9% of its axial stiffness compared to the QI laminate for a 23.0% improvement in 9.9% reliability load. Hence, the greater design space of tow steering provides the RTS design greater flexibility to obtain high imperfect buckling loads with stiff axial response.

The manufactured RTS cylinder, ${\mathrm{RTS}}_{\text{man}}$ has similar ${\hat{P}}^{99.9}$ and E values to the optimizer-found solution, ${\mathrm{RTS}}_{\text{1}}$. ${\mathrm{RTS}}_{\text{man}}$ was a laminate design with a high fitness value in a dynamic-imperfection optimization [17] based on a random combination of the first 20 eigenmodes of the QI cylinder. In each generation, the weighting of eigenmodes was changed to ‘dynamically’ vary the imperfection and incorporate a first-order robustness analysis. The optimization function maximized the imperfect-geometry buckling load of the RTS laminates with the eigenmode-affine imperfection signature. Despite the higher-wavenumber QI eigenmodes not being representative of composite cylinders, which are actually dominated by low-wavenumber modes [35], the dynamic optimization converged on a relatively robust layup that has a high ${\hat{P}}^{99.9}$, albeit with a slightly lower E as this was set to 90% of the QI stiffness during this optimization.

**Figure 9.**
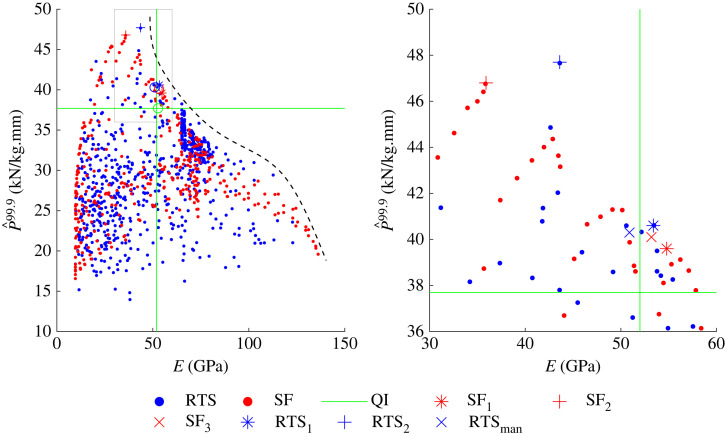
Data from RTS (blue) and SF (red) optimizations. QI data are shown as green lines for ${\hat{P}}^{99.9}$ (*y*-axis) and E (*x*-axis), respectively. Layups tabulated in table 2 are plotted with different markers. Note the optimization analyses found solutions that have ${\hat{P}}^{99.9}$ and E greater than the eight-layer QI. Boxed region is shown on the right-hand side as zoomed-in figure. (Online version in colour.)

To investigate the trade-off between ${\hat{P}}^{99.9}$ and E further, RTS and SF data from both optimizations are plotted on a single plot, as shown in figure 9. The green lines represent the QI data for ${\hat{P}}^{99.9}$ (*y*-axis) and E (*x*-axis), respectively. Of the laminates analysed, there are many layups that have greater ${\hat{P}}^{99.9}$ than the eight-layer QI cylinder, but have lower axial stiffness. Many SF and RTS laminates have a greater E than the QI cylinder, but often to the detriment of ${\hat{P}}^{99.9}$. Only 13 of the 1261 layups investigated have a greater ${\hat{P}}^{99.9}$ and E than the eight-layer QI cylinder, indicating that a trade-off is necessary to achieve one over the other. Indeed, the results suggest a Pareto front generated by the data, as shown by the dotted black line in figure 9, which indicate possible optimal combinations of ${\hat{P}}^{99.9}$ and E.

## 6. Conclusion

On account of the susceptibility of thin-walled cylinders to buckle prematurely due to initial imperfections, VAT composites have been investigated as a potential avenue for alleviating imperfection sensitivity. While previous research has optimized the layup of variable-stiffness cylinders for perfect-geometry buckling, the present research offers a novel methodology by including realistic imperfections within the optimization and optimizing the fibre angles for imperfect-geometry buckling load. The imperfections included in the optimization are taken from a measured dataset and are therefore realistic manufacturing imperfections. To offer a statistically significant estimation of robustness, the FOSM methodology of estimating the probability distribution of buckling loads as a function of this measured dataset was implemented within the optimization. Using the Mahalanobis transformation and spectral decomposition, it was possible to reduce the number of analyses needed considerably to calculate statistical features of the estimated distribution. The 99.9% reliability point on the estimated reliability curve was optimized, as it represents a realistic lower-bound for cylinder design (1 in 1000 failure rate). Due to the fibre-angle-thickness coupling of RTS laminates the 99.9% buckling load was normalized by mass and thickness to allow for a fair comparison with constant-thickness designs. Furthermore, an axial stiffness penalty was included within the optimization to ensure that the optimized buckling load of a design is not to the detriment of other mechanical properties.

Due to the optimization maximizing the thickness-normalized 99.9% buckling load and not minimizing mass by varying the number of plies, the mass of the RTS cylinders is always equal to or greater than any SF counterpart. Therefore, an optimization that minimized the mass while keeping the thickness-normalized buckling load constant would be another approach in showing the benefits of RTS over conventional SF layups. If the mass-normalized 99.9% imperfect-geometry buckling load of ${\mathrm{RTS}}_{\text{1}}$ is compared against the mass-normalized 99.9% imperfect-geometry buckling load of the eight-layer QI cylinder, the RTS cylinder exceeds the QI by 103%. In addition, the analyses carried out within the present work do not consider the multitude of features that a thin-walled cylinder may require when in an industrial application such as inspection holes, cutouts, pad ups and brackets. RTS allows tailoring around these components to minimize the disruption to the stress field, further decreasing the sensitivity to imperfections.

The thickness-normalized 99.9% reliability load of both SF and tow-steered cylinders were optimized within the present study and compared to the state-of-the-art and industry-standard laminate, an eight-layer QI laminate. When the axial stiffness penalty was applied, the optimized SF and tow-steered designs had a 6% and 8% greater thickness-normalized 99.9% buckling load than the eight-layer QI cylinder, respectively. When the axial stiffness penalty was relaxed, the thickness-normalized 99.9% buckling load of the optimized SF cylinder exceeded the eight-layer QI cylinder by 23% for a 32% drop in axial stiffness compared to the QI. Without the axial stiffness penalty, the optimized tow-steered design exceeded the thickness-normalized 99.9% buckling load of the eight-layer QI cylinder by 37% for a 17% drop in axial stiffness. These results indicate that the tow-steering manufacturing technique used within the present study successfully increases the design landscape to allow a more favourable trade-off between stiffness and imperfect buckling load.

Despite the convergence on high-reliability laminates, some limitations of the present work offer interesting avenues for further research. To address the time and computational cost of numerous analyses, it is suggested that surrogate modelling [59] would be an efficient methodology to pursue. Presently, the 99.9% reliability point has been used in the objective function with the assumption of normally distributed imperfect buckling loads. Further research into other reliability points (95%, 99%) could be of interest for less safety-critical structures and, if appropriate, assuming different distributions. For example, extreme value distributions generally govern the statistics of subcritical buckling phenomena [8]. Finally, the highly reliable SF and RTS laminates optimized herein present promising layups that could be manufactured to verify their performance.

## Data Availability

Data are available at the University of Bristol data repository, data.bris, at http://dx.doi.org/10.5523/bris.19580ywiofnuu26i8u4zd9ixxu [60].

## Footnotes

1 Only 2 of the 50 laminates tested in the $1\times 10^{-3}$ step-size analysis were less than 0.995 of the $1\times 10^{-15}$ step-size analysis.

2 The QI laminate has a layup of ${\left[\pm 45,0,90\right]}_{\text{s}}$, and the 48-layer QI laminate has a layup ${\left[0,-45,90,+45,0,-45,+45,90,-45,90,+45,+45,90,0,0,90,+45,-45,+45,-45,0,0,90,-45\right]}_{\text{s}}$.

3 The material differences, while present, are not as large as both lamina material are high-performance pre-preg.
